# Climatology

**Published:** 1879-02

**Authors:** F. C. Schaefer

**Affiliations:** 90 Warren Ave.


### ARTICLE III.

CLIMATOLOGY. By F. C. SCHAEFER, M. D. An abstract of a paper read before the Chicago Medical Society, Nov. 18, 1878.

In considering the subject of the climatology of any given health resort, it would seem necessary to inquire into the natural constituents of the atmosphere, together with the modifying influences brought to bear upon it through the diurnal rotation of the earth upon its axis, the inclination of the ecliptic, the lay of the land, the contour and altitude, the quality of the soil, the varying state of the air as to temperature, humidity, rainfall, dryness, its electric and ozonic conditions, prevailing winds, and proximity to bodies of water, together with the innumerable impurities, organic and inorganic, that enter it from various causes. The subject thus becomes at the outset complicated and almost unlimited in its scope. In the present paper I propose to consider the most important merely of the topics suggested under the following heads: Temperature; Humidity; Dryness; Rain Fall; Winds; Vegetation; Contour of Land; Nature of Soil; Sea Air; Altitude; Ozone; Electricity, and Atmospheric Impurities.

TEMPERATURE.—The influences exerted by the temperature of the atmosphere upon the human organism, are not fully understood, but authorities agree in a general way upon the following points:

I. *Very warm temperature* (20° upward) produces a debilitating and relaxing effect upon all the tissues, diminishing vital affinity. Thus occurs the lassitude of dwellers in tropical climes, and in our own climate when the thermometer remains above 20 degrees for any length of time.

The pathological sequences of great warmth manifest themselves in passive congestion of the brain, lungs, liver, kidneys and spleen, with softening of the heart and intestinal fluxes. Upon the latter point I refer to Dr. N. S. Davis' meteorological report to the American Medical Association of 1876.

II. *Moderately warm, dry, and moderately cold, dry climates* act as tonics, modified by individual idiosyncracy.

III. *Very cold temperature* is sedative, producing a decidedly soporific effect. The experience of Dr. Kane and the Arctic explorers confirms this.

IV. *Frequent diurnal changes* from warm to cold, and *vice versa*, are most pernicious, and produce the phenomena, so frequently noted, of a "common cold."

V. *Equable temperature.*—All medical authorities agree that an equable temperature, all things considered, is the best calculated to assist in the recovery of invalids, and this seems to be the chief condition in selecting a climate for patients suffering from any form of disease.

VI. From Dr. Chas. Dennison's report to the International Medical Congress, 1876, I deduce the following:

"*Warm, moist climates* in Europe are those recognized as sedative, while *moderately cold climates* are said to be stimulating.

"Heat is opposed to stimulation, so far as the nervous system is concerned, and this is demonstrated by Eckhart's experiments upon frogs. When the nerves are subjected to a temperatare much above normal, the electrical currents through them are lessened, and at last stop."

VII. *Heat in shade* is said by Pattery (Surg. R. N. of England), to have a sensible effect in increasing the temperature of the body.

WINDS play their part in influencing climates and usually very favorably by freeing the air of infusoria and noxious gases, and lowering temperature in times of great heat; but they are equally liable to carry the former to otherwise healthful climates.

In selecting therefore a climate that may be favorable to any individual patient, we must not neglect to ascertain from what quarter proceed the prevailing winds. A wind blowing through a pine forest of some magnitude, and thence over the point of selection, necessarily carries with it an influence for good.

Again, a wind traversing a desert, afterwards blowing over a health resort, must of necessity cause a drying of vegetation and great evaporation of moisture that will result favorably or unfavorably according to the quality of soil and climate.

Among the disastrous winds of this nature may be mentioned the simoon of the Orient.

That winds purify the atmosphere of infusoria is abundantly attested by the fact that epidemics frequently diminish in intensity and disappear after wind storms, and by the comparative immunity from malarial disease, enjoyed by people living in a locality where all circumstances favorable to producing malarial fevers are present.

DRYNESS is regarded as *par excellence* the essential feature of an atmosphere beneficial for scrofula, rheumatism, catarrhal inflamation, kidney diseases, laryngitis, neuroses; and dryness in a clear climate containing no dust, is said to be decidedly beneficial in tuberculosis; but dryness where considerable dust accumulates and impregnates the atmosphere, is deleterious in proportion to the quantity of dust present. The particles of dust, it has been asserted, are frequently the active agents in producing phthisis, through an irritating effect upon the lung tissue—their accumulation here setting up a nidus of destructive inflammation, besides supporting septic germs. Prof. Tyndall has shown that dust-laden air is necessary to the existence of these organisms.

HUMIDITY is regarded differently by different authors, but statistics show that the most healthful climates as a rule, contain continuously not more than about Gms. 0.65 of moisture to the cubic foot of air.

It has been shown that in health resorts, where incipient phthisis, rheumatism, neuroses and visceral diseases improve rapidly, e is a minimum of humidity in the atmosphere, and, in several of the most noted, the constant humidity does not exceed Gms. 0.53 to the cubic foot, when there is no rain.*

Unfortunately, we have insufficient data upon which to base positive statements; most of the records upon this subject being only relative.

Great humidity interferes with cutaneous exhalation, and produces a congestion of the viscera. This state of the atmosphere causes a general depression of the system, especially if accompanied by a high temperature. The air being loaded with moisture, there occurs a correspondingly slight evaporation from the surface of the body and its temperature is kept at its maximum.

RAIN FALL.—Rain, it is claimed, cleanses the atmosphere by washing impurities to the ground and carrying them away, when drainage is good.

The electrical disturbances accompanying rain storms are said to increase the quantity of free ozone.

The presence or absence of rain influences various climates differently, according to certain local causes, and a knowledge, simply, of the number of rainy days per month, or during the year, gives one but an imperfect idea of the character of the permanent climate; hence it will not do to be governed by the rain gauge alone in selecting a resort. What might be a surfeit of rain for one spot would prove an insufficiency for another. Considerable rain at an inland point, where the drainage is unusually good, is often of more value to the climate than the same or even a lesser quantity of rain to land on the windward side of a large body of water or upon an island in the sea, where vegetation is supplied with moisture by the more constant humidity of the air.

Again, rain storms, distributed through the year, giving a reasonable number of rainy days to each month, are *generally* more serviceable to the healthfulness of a climate than immense rain storms, with long intervals of dry weather, as may be readily understood.

In California, for instance, where no rain falls for five or six months of the year, the dust that accumulates in the air, in many localities, is so intolerable that it affects to a great degree the

* Proceedings of Int. Congress, '76. Dr. Denison.

good quality of dryness. The dust is undoubtedly frequently the exciting cause of disease; besides, the eyes of an invalid grow weary in looking upon the vast expanse of desert-like territory—nature's mantle of green being frequently entirely absent for months. Oculists would be very unwise in sending their patients to such localities.

VEGETATION plays an important feature in modifying climate, and does so by increasing the amount of rainfall.

All trees give off an abundance of oxygen or ozone and consume carbon di-oxide.

Forests intercept germs migrating through the atmosphere. The generated ozone, according to Prof. Schroeder, unites chemically with many of these conceded disease producers, destroying them. Prof. A. L. Loomis, in his recent address before the American Medical Association at Buffalo, referred to this subject in the following terms: "For some years pulmonary invalids have been recommended to take up their abode in the midst of pine forests. It has been known that they did well amid such surroundings, but why they did well has been an unanswered question. The more extensive and primitive the evergreen forests, the better adapted is the climate, to phthisical patients. The turpentine exhaled from these pine or hemlock forests, possesses, to a greater degree than any other known substance, the power of converting the oxygen of the atmosphere into ozone, thus rendering the air very pure, and consequently antagonistic to phthisical development. Experiment has shown that the direct inhalation of ozone has little, if any, power in preventing or arresting the progress of phthisis. We must, therefore, conclude that it is not the action of ozone upon the respiratory surfaces that renders the climate of localities where it is found in excess, especially salubrious, but that by its power of destroying noxious gases and atmospheric germs, the air is rendered so pure that its action is favorable upon the respiratory surfaces of those predisposed to phthisis."

Among unfavorable effects traceable to vegetation may be mentioned the development of vegetable (disease bearing) spores, which impregnate the air in many localities as the result of vegetable decomposition in stagnant pools and marshes.

Vegetation modifies *temperature*, as shown by M. Becquerel, who, after making 1,400 observations, wrote: "Trees lower temperature by preventing the solar rays from acting upon the soil. The leaves cause the evaporation of large quantities of water by virtue of their organic activity, and increase the superficies liable to be rendered cold by radiation.

In summer the mean temperature of the air, ouside of woods, is higher than inside. In winter the reverse is true. The difference between the mean annual temperature at several miles from wood-land and that inside a wood, is about 3°."

CONTOUR OF LAND.—Local effects are exerted upon climates by unevenness of the earth's surface. Large mountain ranges greatly influence the course of winds. In North America the ranges running in general north and south, the winds blow in these directions, warm from the equator and cold from the polar region; while in Europe the main course of the great ranges being from east to west, the continent is more or less protected against the cold north winds, and hence the mountains of the south of Europe act as strong bulwarks against the excessively warm dry winds of Sahara, which are further modified by evaporation from the Mediterranean. These facts produce wonderful effects upon the climates of immense tracts of country; and to the position of mountain ranges, together with the direction of ocean streams and trade winds, are attributed, in great part, the differences in the climates of Europe and America, with reference to equability in temperature.

SOIL.—The nature of the soil increases or diminishes the humidity of the air. A modern climatologist* has written: "The peculiar dampness which acts most unfavorably upon phthisis, is not usually present where there is the greatest amount of rainfall, nor is it present because large bodies of water are in close proximity; but it mainly depends upon the nature of the soil. To avoid this dampness the soil should be porous and sandy, a loam soil of sufficient porosity to permit the rapid filtering of water from its surface, so that after a heavy rainfall the surface will soon become dry. All clay soils drain slowly and imperfectly, and the peculiar dampness arises which acts so unfavorably

* Prof. A. L. Loomis.

on phthisical patients. Lænnec states that the dampness arising from such a condition of soil, is one of the most certain developing causes ot consumption, and makes mention of a locality having such a soil, in which the dampness was so constant and of such a character, that more than two-thirds of the resident population died of phthisis."

Low soil of certain table lands and valleys, designated as marshy, and river bottoms, are very deleterious to health ; holding, as they do, decayed vegetable matter in solution, which, when favorable conditions of the atmosphere are present, impregnate it with spores of various species giving rise to innumerable disorders, especially malarial fevers.

SEA AIR.—The atmosphere above the sea has a more equable temperature as a rule than the land air, owing as meteorologists inform us, to the following reasons:

I. "Because the rays of the sun penetrate the ocean to a considerable depth, and therefore produce less effect upon the surface."

II. "Because by the agitation of the sea there is a perpetual mingling of the surface with the lower strata."

III. "Because water has a much greater capacity for heat than the dry earth."

The physical forces acting in this manner, the surface of the sea is naturally less readily heated than the earth, and again cooling of the surface proceeds more slowly, because, as the heat radiates from the surface, the specific gravity of the upper stratum is increased, and it must make way for the warmer from beneath. The processes of heating and cooling being thus gradual, they result in materially diminishing the diurnal variation of temperature. The daily variation is placed by standard works on meteorology, at 2° in the torrid zone and 4° to 5° in the temperate zone. The annual range varies from 10° to 24° as one proceeds from the equator towards the polar regions, which is much less than in corresponding latitudes upon land.

The variation in temperature being so slight from summer to winter, it follows that places controlled by a marine climate, must have a comparatively equable temperature.

The sea air is said to be denser than land air generally, and to contain an excess of ozone with consequent freedom from germs; it is therefore stimulating and invigorating, hastening the process of tissue metamorphosis.

The atmosphere being denser, there does not arise the necessity for exercise, (as we shall find in the case in high altitudes), in order to obtain a full supply of oxygen to sustain physiological processses.

There is greater humidity in sea air than in that of the mountain; but the saline element entering the former is claimed to counterbalance, in great part, the effects of humidity. M. Thaon of Nice asserts, however, that sea air is injurious to the rheumatic and dangerous for those having laryngitis and diseases of kidneys.

HIGH ALTITUDES.—The atmosphere of high altitudes is claimed to be purer, to contain less germs and more free electricity and ozone than that of the low lands. Prof. Loomis of N. Y., believing that the purity of the air in high altitudes might be the all important restorative agent in phthisis, says: "When I speak of the purity of the atmosphere, I mean not only its freedom from what are ordinarily called impurities, but its freedom from atmospheric germs. Prof. Tyndall has shown by actual experiment that the air as we ascend becomes freer and purer from those atmospheric germs. In the presence or absence of these organic substances we have a very important element of difference between the air of the low lands and the air of the mountains.

The air of high mountains is very rich in ozone. Dr. Schrœder states that ozone has the power of purifying the atmosphere—purifying it of organic substances; that its purifying power depends upon its oxidizing power; that while oxygen requires a considerable degree of heat before it will combine with other substances, ozone will do so at ordinary temperature. Ozone therefore destroys the products of decomposition by chemically uniting with them. The presence of ozone in the atmosphere is presumptive evidence that it contains no organic substances.

Dr. Chas. Denison, of Colorado, in his comprehensive report to the International Congress, at Philadelphia, 1876, claims that the electric tension of the air increases with altitude. He asserts further "that the diathermacy increases with elevation, there being one degree greater difference between the temperature of shade and sun for each rise of about 220 feet," which he proves by experiment. The humidity decreases as the diathermacy increases. "The sun's rays being less obstructed, there is a proportionately longer influence of sunshine in each 24 hours, besides an additional excess due to a usually cloudless sky over such plains as those east of the Rocky mountains."

According to Humboldt there is a decrease in temperature of about one degree to each 300 feet of elevation.

HIGH ALTITUDES; THEIR INFLUENCE UPON MAN. The experience of scientists and travelers in the regions of great elevation is thus summarized in M. Flammarion's work on meteorology:

"*Breathing* is accelerated, impeded and becomes laborious. There occurs extreme dyspnœa after the least movement.

"*Circulation;* palpitation, acceleration of pulse, beating of carotid, sensation of plenitude in vessels, and frequent hæmorrhages.

"*Innervation;* painful headaches, irresistible desire to sleep, dullness of senses, loss of memory and moral prostration.

"*Digestion;* thirst, strong desire for cool drinks; distaste for solid foods, nausea, eructations and vomiting.

"*Functions of Locomotion;* pain more or less severe in knees and legs; locomotion causing great fatigue and exhausting all strength."

It follows from the preceding observations that high altitudes cause injury to persons suffering from: I, Disease of the heart. II, Diseases of blood vessels, as atheroma. III, Phthisis ulcerosa. IV, Individuals whose vital processes are too rapid. In some of the diseased conditions mentioned above, patients are unfavorably affected on mechanical principles, the organs mentioned being too feeble in their unhealthy state to resist the increased tension occasioned by the diminished atmospheric pressure; but they are also materially modified by increased tissue metamorphosis.

The following named pathological manifestations are considerably relieved, and in many instances almost eradicated by conditions present in high altitudes: I, Phthisis incipiens. II, Scrofulosa. III, Kidney diseases. IV, General atonic condition of entire organism.

Such abnormal states are remedied owing to the great purity of the atmosphere, the diminished pressure, abundance of free electricity and sunshine and the necessity of exercising in order to oxygenize the blood. The atmosphere in higher elevations, containing a less amount of oxygen to a given bulk of air, it becomes necessary to inhale a larger quantity of air in order to obtain a sufficiency of oxygen to exist. This of necessity causes deeper inhalations, distending the lungs to their utmost capacity, in due proportion developing these organs, together with the entire mechanism required for the respiratory function. The natives of Peruvian mountain regions are noted for their lung capacity, and their chests are said to measure considerably more than those of natives of low lands everywhere; and the same is recorded of the aborigines of the Arizona mountains.

Theoretically, and to a very great extent practically, we can safely say that the higher altitudes, with their concomitant conditions, are beneficial in the class of diseases already mentioned, and likewise in all such as require tonic treatment, together with pure atmospheric surroundings, not complicated by the pathological elements already alluded to.

I have thus endeavored to call attention hastily, to the more important elements in every climate, in order to suggest what effect the individual elements and different conditions brought to bear upon these elements, *per se*, may exert upon disease. While it is very well to consider such a subject in this abstract manner, the fact should not be overlooked that we can not prescribe this or that element separately as we might a simple drug. And herein, it seems to me, can be found the cause of so many diverse ideas and vague expressions relative to the kind of climate best adapted to various individuals. One physician orders a change to a high altitude, another to the low lands, a third prescribed sea air to all patients regardless of stage or character of disease, while still another recommends a warm climate, and so on *ad infinitum*. The difficulty in the past has been that our predecessors were eager to attribute the curative results to some one element, not bearing in mind that the same element is variously modified in different localities by local circumstances or general causes.

The empirical methods pursued in selecting climate, were of such a nature as, in many cases, to result in the defeat of the object in view, no account having been taken of the different stages or various forms of the same malady. Phthisis being pre-eminently benefited by a change of climate, this was the first disease of which we have authentic records subjected to climatic theory. That consumptives could be improved in health in this manner, appears to have been known by the profession for fully 1800 years, and can be even traced to Celsus who, at the commencement of the Christian era, advised change of climate for phthisical patients, *providing* their strength would permit, preferring change from a rare to a denser atmosphere. Even to-day the great bulk of medical literature upon climatology is devoted to the treatment of phthisis. Fortunately, however, so far as understood at the present time, the general attribute of the climates beneficial for this dreaded malady, are almost universally of such a nature as to prove more or less useful for many pathological conditions of the human organism.

It is only through experience and long observation on the part of the more thoughtful that we have at length been led to the study of climatology, and are now beginning to recommend changes rationally based upon differential diagnosis and meteorological variations, and doubtless in a very few years there will be less conflicting ideas upon this subject.

It is needless to state that an ideal climate is not to be found for any malady, but after the therapeutic bearing of the disease—conditions to be considered in each case—their modifying influences upon each other, together with their individual and combined effect upon the human body, are once clearly understood, the physician will be much better prepared to act in each case with intelligence and proper foresight. Knowing that he has to deal with ulcerative processes in the lungs, and appreciating the fact that a high altitude is injurious chiefly on account of its stimulating effect upon the arterial system, due to diminished atmospheric pressure, he is prepared to act accordingly. He will advise a lower region, of equable, moderate temperature, of pure air, with minimum humidity, having considerable sunshine, electric tension, favorably located for drainage and for protection against injurious winds. All experience proves that these elements enter largely into a climate beneficial for cases of phthisis ulcerosa. Here credit can be given to no one feature, but to a combination of circumstances and conditions.

Turning to phthisis incipiens, we discover a stage of a disease which can be retarded and possibly entirely relieved by proper climatic conditions. The following qualities produce, according to statistics, very favorable results: a reasonably high altitude, say 2,000 to 6,000 feet, with pure atmosphere, of equable temperature, containing considerable free ozone and electricity, with moderate dryness. This state of climate is recommended in a majority of cases of phthisis incipiens, general atony, neurosis and diseases of the kidneys and other viscera. There are, however, invalids for whom sea air is preferred, viz.: those who are incapable of taking muscular exercise, and yet whose processes of tissue change should be hastened. These are generally middle aged and old people.

### CONCLUSIONS.

If we note carefully the observations of students of climatology, with reference to their bearing upon disease, we find the most healthful climate, as a rule, contain the following mentioned qualities, modified by the conditions already mentioned, the degree of superiority being governed by the number and grade of the latter elements present: equable temperature, moderately warm or cold; moderate dryness; an abundance of sunshine, strong electric tension and considerable free ozone; sufficient winds to assist in removing impurities; contour of land and nature of soil favorable for prompt drainage; moderate rain falls; the presence of bodies of water; and proximity to forests.

For a limited class or special stage of diseases we find strongly recommended: high altitude; warm or cold temperature; sea-air; moderate humidity.

And finally, points regarded as universally injurious are: decided humidity; lowness of lands, especially if the soil is marshy, or of a clayey nature; *negatively*, the absence of the abovementioned better attributes.

90 WARREN AVE.

